# Two-Year Follow-Up of Humoral and Cellular Immune Responses to SARS-CoV-2 in Healthcare Professionals

**DOI:** 10.3390/vaccines13111163

**Published:** 2025-11-14

**Authors:** Silvie Ostřížková, Jan Martinek, Denisa Budirská, Hana Zelená, Alena Kloudová, Eduard Ježo, Rastislav Maďar, Hana Tomášková

**Affiliations:** 1 Department of Epidemiology and Public Health, Faculty of Medicine, University of Ostrava, 70300 Ostrava, Czech Republic; 2 Public Health Institute in Ostrava, 70200 Ostrava, Czech Republic

**Keywords:** COVID-19, SARS-CoV-2, interferon-γ, virus neutralization test, immunoglobulin G, vaccination

## Abstract

Background/Objectives: Following the global spread of SARS-CoV-2, there was an urgent need for vaccine development to support immune protection. This study aimed to evaluate the impact of active and hybrid immunity on the durability of immunoglobulin G (IgG), neutralizing antibodies, and cellular immune responses over a two-year period. Methods: This longitudinal study was conducted from February 2021 to December 2023 at the Public Health Institute in Ostrava, Czech Republic. Anti-S IgG was measured using ELISA (Euroimmun), neutralizing antibodies via an in-house virus neustralization test (VNT), and cellular immune response using the IGRA test (ELISA, Euroimmun). Participants also completed a questionnaire on demographics, COVID-19 history, symptoms, and vaccination. Statistical analysis included descriptive and non-parametric tests (Mann–Whitney U, Kruskal–Wallis) at a 5% significance level. Results: The cohort included 149 individuals, 97.3% of whom were vaccinated with Comirnaty (Pfizer/BioNTech). A total of 17% had confirmed infection prior to vaccination and showed up to two-fold higher neutralizing antibody levels (*p* < 0.001) within 2–6 weeks postvaccination. Postvaccination infection was reported in 35% of participants. Although antibody levels declined over the 2–100 week period, participants remained seropositive across all three parameters. Cellular immune response (interferon-γ) remained consistently high throughout follow-up. Conclusions: The study demonstrates long-term durability of IgG and neutralizing antibodies and confirms durable cellular immunity up to two years postvaccination. Hybrid immunity significantly enhanced neutralizing antibody levels, supporting its added value in protective immunity against SARS-CoV-2.

## 1. Introduction

SARS-CoV-2 (Severe Acute Respiratory Syndrome Coronavirus 2) is a coronavirus with 87% genetic homology to the SARS-CoV-1 virus [1]. The virus has primarily evolved toward variants with lower pathogenicity but higher transmissibility, although it can be stated with near certainty that it will continue to mutate [2].

The course of COVID-19 is the result of a combination of the intrinsic properties of the virus and the host immune response. Moreover, the severity of infection is significantly influenced by risk factors such as senescence, obesity, chronic respiratory and cardiovascular diseases, cancer, or diabetes mellitus [3].

The host immune response acts through both the innate and adaptive components. Innate immunity forms the first line of defense and, in some cases, can eliminate SARS-CoV-2 without activation of the adaptive component, utilizing physical barriers, phagocytic cells (e.g., macrophages and neutrophils), cytokines, and complement. In other cases, however, inappropriate activation of immune defenses may occur [2,4].

Within a few hours after the virus enters the host, a rapid immune response is initiated, mediated by type I and III interferons (IFN I/III), cytokines (IL-1, IL-18, IL-6), and chemokines (chemokine ligand 2 and 7). These molecules also contribute to the inhibition of viral replication [5]. Excessive cytokine production may result in a cytokine storm, which can lead to pulmonary edema and acute respiratory distress syndrome (ARDS) [6].

Although COVID-19 is primarily regarded as a respiratory disease, systemic (extrapulmonary) manifestations are frequently observed, attributed to the widespread expression of ACE2 (angiotensin-converting enzyme 2), which may lead to multiorgan involvement. These systemic manifestations arise from an inflammatory syndrome and result in elevated levels of inflammatory chemokines, alarmins, and serum interleukin-6 (IL-6), profound lymphopenia, coagulopathy, direct infection of pulmonary capillary endothelial cells, or hyperactivated innate immune defenses [7,8].

Unlike innate immunity, the adaptive component is characterized by its ability to recognize specific antigens through receptors on B and T lymphocytes and to generate immunological memory, making it faster and more effective upon re-exposure to the antigen [4]. CD4^+^ and CD8^+^ T lymphocytes play a key role in contributing to long-term protection and mitigation of severe disease [9]. SARS-CoV-2 infects alveolar macrophages, which produce chemoattractants that recruit T lymphocytes. These subsequently produce IFN-γ, which stimulates the release of pro-inflammatory cytokines and enhances the activation of the adaptive immune response [10].

A critical component of the adaptive response is humoral immunity, which complements the function of antibodies and includes complement, ficolins, pentraxins (e.g., C-reactive protein, CRP), and collectins such as mannose-binding lectin (MBL), which binds and inhibits SARS-CoV-2. Various haplotypes in MBL2 are associated with differences in disease severity. MBL can activate the lectin pathway of complement [2,11]. Approximately 1–2 weeks after the onset of the first COVID-19 symptoms, immunoglobulins A, M, and G (IgA, IgM, IgG) develop rapidly, with IgG levels continuing to rise and persisting for more than a year [12,13]. Their levels also correlate with disease severity and age [14,15]. The presence of IgM indicates acute infection or recent recovery [16]. IgA is the main effector molecule of mucosal immunity and helps protect the host from viral attachment and invasion [3].

The combination of prior infection and vaccination generates hybrid immunity, which provides even stronger protection. Several vaccination strategies have been developed against SARS-CoV-2, with mRNA and adenoviral vector vaccines being the fastest deployed and most widely used, aiming to stimulate both humoral and cellular immune response [9]. Depending on the number of doses administered, the effectiveness of immune protection is estimated to reach up to 96% and to persist for more than 5 months. After two or three doses, protection against critical or fatal outcomes exceeded 97% [17].

The aim of this study was to analyze the humoral and cellular immune responses among employees of the Public Health Institute in Ostrava (PHI Ostrava) who either experienced SARS-CoV-2 infection or were vaccinated against this infection. Changes in antibody levels over a two-year period and the overall seroprevalence among employees with and without a history of infection were systematically monitored.

## 2. Materials and Methods

### 2.1. Study Population

As part of a prospective study on the immune response to SARS-CoV-2 among employees of the Public Health Institute Ostrava (PHI Ostrava), Czech Republic, data were collected from a total of 287 participants, of whom two withdrew from the study due to retirement. PHI Ostrava is a regional public health authority comprising several workplaces located in different parts of the Czech Republic, including the main office in Ostrava and a branch in Ústí nad Orlicí. Employees from all affiliated workplaces were invited by email and were asked to indicate their consent or refusal to participate in the study. Written informed consent was subsequently obtained from all participants. The study was approved by the Ethics Committee of PHI Ostrava under protocol number P01/2021.

Participants were divided into three groups according to their infection and vaccination status at the beginning of the study.

- Group 1 (vaccinated group): Individuals who completed the full primary COVID-19 vaccination series (two doses), or who had recovered from the disease and were subsequently vaccinated (*n* = 149).
- Group 2 (infected group): Individuals who had experienced COVID-19 infection and had not been vaccinated prior to the start of the study (*n* = 76).
- Group 3 (uninfected/unvaccinated group): Individuals who were neither vaccinated nor reported a history of COVID-19 infection (*n* = 60).

**Figure 1:**
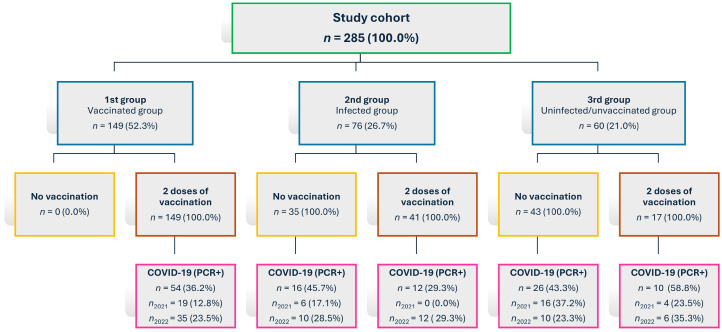
Flowchart showing cohort distribution and changes in infection and vaccination status during the study period; *n* = number of participants.

During the study, additional subgroups were created based on vaccination or COVID-19 infection occurring during the follow-up period. Individuals in Groups 2 and 3 were later affected by vaccination and/or COVID-19 infection (as seen in Figure 1). Given these circumstances, only the cohort of individuals from Group 1 (vaccinated group) is described here, as this group was selected for its highest level of homogeneity.

Participants completed a questionnaire containing demographic data (age, sex, level of education, and job position), medical history (including the presence of chronic diseases and current health status), and information related to psychological well-being. Data on COVID-19 testing were also recorded, including the date and results of PCR or antigen tests, clinical symptoms, duration of illness, and the course of recovery. Vaccination records included the dates of administration of each dose, including booster doses.

Each participant provided a venous blood sample at their workplace site. Specifically, a 5 mL tube of blood was collected for the determination of IgG, IgM, and IgA antibodies using ELISA and VNT methods, and a 2 mL tube of anticoagulated blood was collected for the assessment of cellular immune response. The planned sampling schedule included time points at 1, 3, 6, 12, 18, and 24 months after enrollment in the study. In the event of reinfection, a sample was obtained 3 weeks after recovery and 2 weeks after vaccination, followed by sampling according to the original schedule (Figure S1). Due to changes in the epidemiological situation, the study was conducted from February 2021 to December 2023.

### 2.2. Methods

For the determination of immunoglobulins, the anti-S IgG ELISA method (Euroimmun) was used in accordance with the manufacturer’s instructions. The positivity index (PI) was used for evaluation. Results > 1.1 were considered positive, results < 0.9 were considered negative, and values between 0.9–1.1 were classified as borderline results. Results were later also reported in international units (BAU/mL); however, for the sake of consistency, PI continued to be used in this study as the primary parameter for determining positivity.

For the determination of neutralizing antibodies, the virus neutralization test (VNT, in-house method) was employed as the gold standard. Samples were serially diluted in two-fold steps. Final concentrations reported as 1/10, 1/20, etc., were expressed as the inverse of the highest dilution still inhibiting the cytopathic effect of the virus by at least 50%. A neutralizing titer of 0 was considered negative, a titer of 10 was considered borderline, and a titer ≥ 20 was considered positive. Titers higher than 2560 were reported as “>2560”.

For the detection of the specific cellular response, an interferon-gamma release assay (IGRA) was used. This assay measures IFN-γ production after stimulation of T lymphocytes with SARS-CoV-2-specific antigens, allowing assessment of the functional activity of specific T cells. Values > 200 mIU/mL were considered positive, values < 100 mIU/mL negative, and values between 100–200 mIU/mL borderline. Values higher than 2500 were reported as “>2500”.

### 2.3. Statistical Analyses

Data were analyzed using descriptive statistics. For continuous variables, the median, interquartile range (IQR), arithmetic mean, standard deviation, and minimum and maximum values were calculated. Categorical variables were presented as absolute and relative frequencies and summarized using frequency tables.

To evaluate differences in humoral and cellular immune response levels between groups, nonparametric tests were applied—the Wilcoxon rank-sum test for comparisons of two independent samples and the Kruskal–Wallis test for comparisons involving more than two groups. Correlations between the analyzed immunological parameters were assessed using Spearman’s rank correlation coefficient. Statistical significance was set at a 5% significance level. All analyses were performed using Stata software, version 17 (StataCorp, College Station, TX, USA).

## 3. Results

Group 1 consisted of a total of 149 individuals with a mean age of 49.3 ± 11.4 years (range: 24–78 years). Women predominated in the cohort, representing 85.9% (*n* = 128) compared to men at 14.1% (*n* = 21). The mean age did not differ significantly between men (49.1 years) and women (49.3 years) (*p* = 0.936). Participants were predominantly from the Ostrava workplace (*n* = 97; 65.1%) and the Brno branch (*n* = 26; 17.5%), with the remaining participants recruited from other regional offices across Moravia, Silesia and eastern Bohemia (*n* = 26; 17.4%).

No statistically significant differences in VNT or IGRA values were observed between sexes or across age groups at the first sampling. In IgG values, a statistically significant difference was found in the subgroup of vaccinated individuals without prior COVID-19 infection, where men had significantly higher IgG levels than women (median PI 7.9 vs. 7.0; *p* = 0.031). At the second sampling, this difference was no longer statistically significant (*p* = 0.211). In the subgroup with prior infection before vaccination, no significant sex-related differences in IgG levels were observed (*p* = 0.461). Age was not significantly associated with IgG levels (*p* = 0.069 and *p* = 0.551, respectively).

The most frequently administered vaccine was Comirnaty (Pfizer/BioNTech, Mainz, Germany), given to 97.3% (*n* = 145) of participants. Two individuals (1.3%) received Spikevax (Moderna), and another two individuals (1.3%) received Vaxzevria (AstraZeneca, Cambridge, UK).

The subgroup of individuals who received the first booster dose included 91 participants (31.7%), while three participants (1.0%) received the second booster dose.

**Figure 2:**
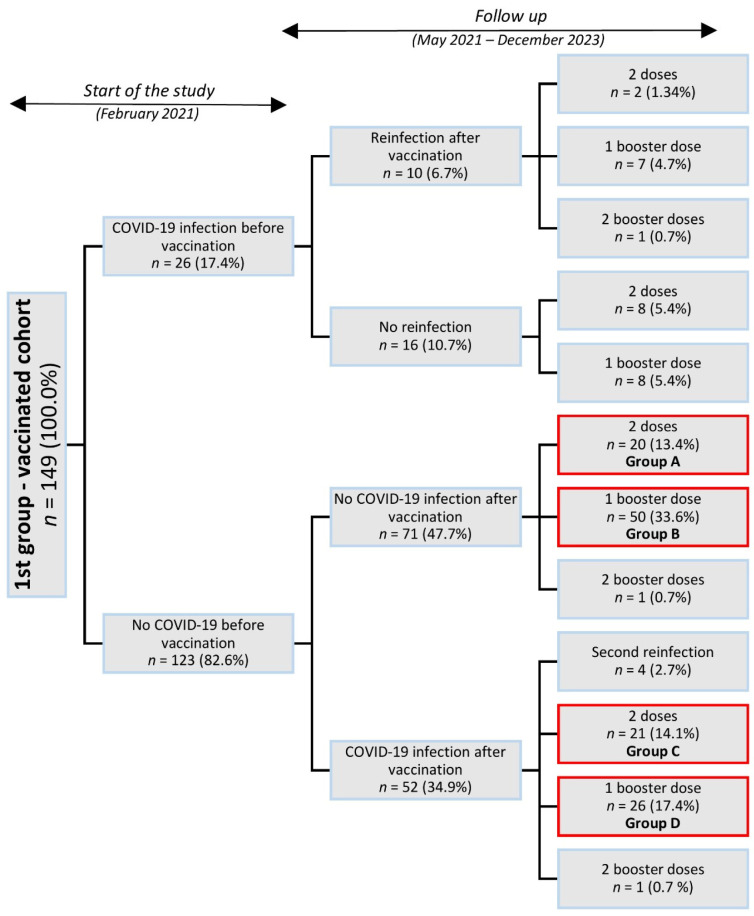
Flowchart of the distribution of participants into groups and subgroups by status of vaccination during COVID-19 infection. Note: The highlighted groups underwent more detailed analyses. Percentages were calculated based on the total sample size (*n* = 149).

Figure 2 shows a diagram illustrating the status of participants within Group 1. In this group, 26 individuals (17.4%) reported having had COVID-19 prior to vaccination. A total of 71 participants (47.7%) reported that they had never had COVID-19. In total, 52 participants (34.9%) reported having contracted the disease after vaccination. Participants who had received two primary doses or two primary doses plus one booster dose and who reported either never having had COVID-19 or having had it only after vaccination were selected for more detailed analyses.

Blood samples were collected according to the schedule described in the Participants and Data Collection section. The first samples from participants vaccinated with the first dose of Comirnaty were collected starting in February 2021. The highest number of samples was collected in February 2021. In total, 1181 samples were obtained during the study. As the number of collection time points increased, the number of participants in the study gradually decreased. The final samples were collected in December 2023.

The results were compared between the group that had COVID-19 prior to vaccination and the first blood draw, and the group without a history of COVID-19 before entering the study and thus prior to the first blood draw (Table 1). COVID-19 was diagnosed in a total of 26 participants within a range of 12 to 224 days before vaccination. A statistically significant difference between the groups was observed only in VNT results (*p* < 0.001). Neutralizing antibody titers were up to twofold higher in individuals who had been vaccinated and had recovered from COVID-19 compared to participants who had been vaccinated but had not had the disease.

In individuals who had neither experienced COVID-19 infection prior to vaccination nor received a booster dose (Group A in Figure 2), IgG antibodies, neutralizing antibodies (VNT), and cellular immune response (IGRA) were evaluated at defined time intervals following the administration of two vaccine doses. At the beginning of follow-up, this group included twenty participants (Table 2). For IgG antibodies, a slight increase was observed initially, followed by a decline between weeks 27 and 48 (week 27–32: *p* = 0.016; week 40–48: *p* = 0.017), and a subsequent rise after week 67. VNT titers showed a similar trend, with a marked decrease between weeks 15 and 48 (week 15–20: *p* = 0.182; week 27–32: *p* = 0.016; week 40–48: *p* = 0.017) but without a distinct initial increase. The cellular response measured by IGRA was initially high, then declined, and from week 27 onward showed a gradual increase (week 27–32: *p* = 0.001; week 40–48: *p* = 0.002), approaching the levels observed during the first weeks after vaccination.

The IgG, VNT, and IGRA parameters were evaluated in the same manner for the group of individuals who had received two doses of vaccination and either did or did not develop COVID-19 after vaccination (groups A and C shown in Figure 1). These groups could be monitored up to week 32, after which new infections began to occur among participants. In the group that subsequently contracted COVID-19, lower antibody levels were observed; however, the differences were not statistically significant. The levels of neutralizing antibodies and cellular immune responses showed considerable variability in the results (Table 3).

**Figure 3:**
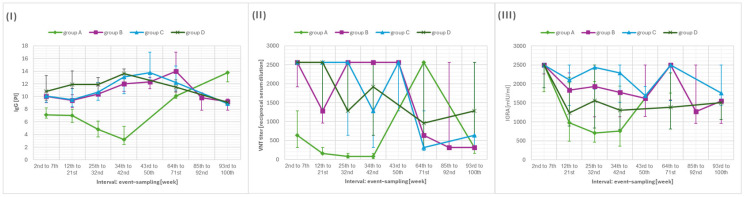
(**I**) Median and interquartile range (IQR) of anti-S IgG levels, (**II**) neutralizing antibody titers and (**III**) interferon-gamma release assay in individuals with different combinations of events—vaccination and postvaccination infection (group A—2 doses of vaccination, B—1 booster dose, C—2 doses of vaccination and infection, D—1 booster and infection). Note: Median values are connected by lines across weeks.

Table 4 compares the levels of IgG antibodies, neutralizing antibodies (VNT), and cellular immune responses (IGRA) between participants who had received two vaccine doses plus a booster dose, those who had received two vaccine doses and subsequently experienced COVID-19 infection, and those who had received two vaccine doses plus a booster dose and experienced COVID-19 (Figure 3).

When compared with the group that had received only two vaccine doses, all other groups showed statistically significantly higher IgG and VNT titers during weeks 2–42 (*p* < 0.001 for all comparisons). For IgG antibodies, a significant difference was also observed during weeks 64–100 (*p* = 0.005 for all comparisons), while for VNT titers, a significant difference was detected during weeks 64–71 (*p* = 0.006).

The dynamics of IgG antibodies show an initial rise following vaccination, followed by a decline and a subsequent increase after week 85 (*p* = 0.005 in both comparisons). Neutralizing antibodies followed a similar pattern, with an initial postvaccination rise, a decline, and a subsequent increase between weeks 64 and 100 (week 64–71: *p* = 0.005; week 93–100: *p* = 0.672). A comparable trend was also observed for the cellular response measured by the IGRA test.

When comparing cellular immune responses, a statistically significant difference between groups was observed during weeks 12–32 (*p* < 0.001 in both comparisons), with groups that had experienced infection and/or received a booster dose showing higher medians than the group without these immunizing stimuli.

**Figure 4:**
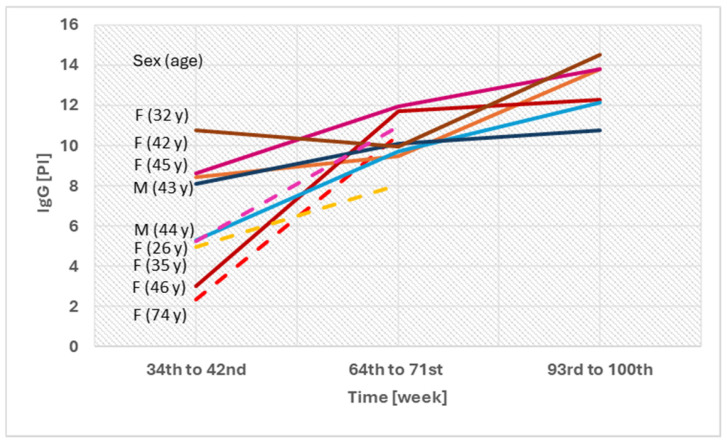
Individuals with two doses of the vaccination and no prior COVID-19 infection—comparison of IgG levels between 34 and 100 weeks after vaccination. F—female; M—male; PI—positivity index; y—years.

In nine individuals who received only two doses of the vaccine, asymptomatic infection was presumed. In these participants, an increase in IgG levels was observed at 34–42 weeks post-vaccination. Most of them also showed elevated VNT and IGRA levels, although these values generally remained high throughout the follow-up period. Only five individuals were available for follow-up at 93–100 weeks. In these cases, an increase was observed at 64–71 weeks, followed by a decline at 93–100 weeks; however, all values remained positive (Figure 4).

Across the full set of analyses, a total of 1181 blood samples were collected; however, due to preanalytical or analytical issues, some measurements were not performed. IgG negativity was detected in one out of 1172 samples (0.1%). Negative VNT results were observed in two cases (0.2%), and borderline results in six cases (0.5%) out of 1177 samples. For IGRA, negative results were recorded in seven cases (0.7%) and borderline results in 22 cases (2.0%) out of 1106 samples. At least one positive parameter was observed in each participant throughout the study.

Correlation analysis performed for the entire dataset revealed statistically significant associations between the analyzed immunological parameters: IgG and VNT (r_s_ = 0.704, *p* < 0.001), VNT and IGRA (r_s_ = 0.409, *p* < 0.001), and IgG and IGRA (r_s_ = 0.307, *p* < 0.001).

Overall, both humoral and cellular immune responses showed a decline over time but remained detectable in most participants up to the end of the follow-up period.

## 4. Discussion

Testing was performed across all variants classified as variants of concern (VOC) (Alpha–Omicron). During the study period covering Alpha to Omicron variants, vaccine effectiveness fluctuated, reflecting changing viral characteristics. During the period of Omicron predominance, however, lower mortality and hospitalization rates were observed, partly attributable to the protective effects of vaccination [18,19]. Nevertheless, questions remained regarding the effectiveness of vaccines and the duration of their protective effect, which was also the focus of our study. However, data on the specific SARS-CoV-2 variants infecting individual participants were not available, and thus no variant-based correlation analysis could be performed.

We assessed both humoral and cellular immune responses to provide a comprehensive characterization of post-vaccination immunity, as these responses may not always correlate in individual participants. The IGRA reliably detects prior infection, especially in vaccinated or immunocompromised individuals, complementing serological testing and improving detection sensitivity [20,21,22].

Among the monitored participants, IgG, VNT, and IFN-γ levels were compared. For neutralizing antibodies, a statistically significant effect of prior COVID-19 infection before vaccination was observed. VNT titers in individuals with hybrid immunity were up to twofold higher within 2–6 weeks after vaccination. Similar findings were reported in a study of healthcare workers from Oregon [23]. No significant effect on the IgG antibody response was observed. IgG responses appear to remain stable even in relation to disease severity [24]. Some studies, however, reported significant differences between these groups not only in VNT titers but also in IgG antibody levels [25]. IFN-γ levels did not show statistically significant differences between these groups in our study, although other studies have reported marked differences [26,27]. A study from the Czech Republic examining immune responses in vaccinated elderly individuals also demonstrated a higher immune response in those with hybrid immunity [28].

The dynamics of IgG, VNT, and IGRA in all groups monitored for more than 48 weeks showed an initial increase after vaccination, followed by a decline and a subsequent rise starting from week 67. This later increase in antibody levels may have resulted from unrecognized reinfections, which could have affected mean values, given the limited number of participants remaining in this group. Only nine individuals received two vaccine doses and had no PCR-confirmed infection throughout follow-up; however, an asymptomatic course is presumed in these cases, as indicated by increased IgG levels after week 64. For the remaining participants, infection occurrence could not be reliably verified due to confounding factors and extended sampling intervals. The U.S. PARIS study, which evaluated unvaccinated healthcare workers monitored until August 2021 (i.e., before the spread of the Delta and Omicron variants), described a similar kinetics of IgG antibodies, although over a shorter observation period of one year [29,30]. A recent Polish study with a comparable female predominance reported a decline in antibody levels within 20 months after vaccination and confirmed the advantage of hybrid immunity in maintaining higher antibody titers [31]. Likewise, Löfström et al. observed an antibody decline within 12 months in a predominantly female Swedish cohort [32]. Our study extends these findings by providing one of the longest follow-up periods reported to date—up to 24 months—and by incorporating the assessment of cellular immunity.

IFN-γ showed remarkably prominent levels across all groups throughout the follow-up period, with no statistically significant differences between participants who were only vaccinated, those who received a booster dose, those who received two booster doses, or those who had experienced COVID-19 infection. However, during the first sampling period (2–6 weeks after vaccination), higher levels were observed in individuals who had been infected with SARS-CoV-2 prior to vaccination. Numerous studies highlight the crucial importance of cellular immune response, partly due to its longer duration of seropositivity [33]. Moreover, T-cell immunity (CD4^+^ and CD8^+^) has been shown to recognize known SARS-CoV-2 variants as late as 6–7 months after vaccination [9], indicating a sufficiently robust immune response despite viral genetic mutations. Although antibody levels decline over time, cellular immune response remains high in vaccinated and/or previously infected individuals for nearly the entire duration of our study.

Taken together, our data confirm the role of hybrid immunity as the most effective and durable protection against severe COVID-19 [34,35]. A study from the United Kingdom examining 600 individuals after primary vaccination demonstrated that, in the absence of infection, antibody decline is more rapid during the first six months after vaccination compared with the period from 6 to 12 months postvaccination. A similar trend of decline was also observed in individuals who experienced infection after vaccination [36]. Our findings are consistent with these results as well as with studies from Norway [37] and Italy [38].

The main factor that may have influenced the results of this study includes the possibility of asymptomatic or unrecognized infection among participants, as mentioned above. These factors, together with possible genetic predisposition affecting the immune response, and the fact that the severity of infection was not considered for individual participants may have influenced the variability of the observed results. Additional limitations include the relatively small sample size (*n* = 149), changes in subgroup composition over time due to new events such as vaccination or COVID-19 infection, and the uneven sex distribution with a predominance of women (85.9%). Consequently, the results should be interpreted with caution, particularly regarding extrapolation to men and older individuals. In some cases, not all parameters could be analyzed due to preanalytical issues (e.g., insufficient blood sample volume). Furthermore, participants occasionally had to be reclassified between groups due to new infections or booster vaccinations, and the study was also influenced by the emergence and spread of new SARS-CoV-2 variants. Despite these limitations, the consistent trends across immune parameters suggest that findings reliably reflect postvaccination immune kinetics in working-age adults.

Both humoral and cellular responses play a crucial role in protection against infection. Evidence exists of a correlation between neutralizing antibody levels and the degree of protection [17]. However, none of the monitored parameters—neutralizing antibody titers, IgG antibody levels, or cellular immune response—are absolute indicators of protection against infection [17,39]. Nevertheless, the combination of these three parameters provides a robust representation of the immune response in individuals.

## 5. Conclusions

This study provides detailed insight into cellular and humoral immune responses in 149 vaccinated individuals, considering prior COVID-19 infection; it also has one of the longest follow-up periods (up to 24 months), offering unique insight into the long-term durability of postvaccination and hybrid immunity. Individuals with hybrid immunity demonstrated significantly higher neutralizing antibody titers within 2–6 weeks after vaccination, confirming the additive effect of infection-induced and vaccine-induced immunity. Over time, IgG levels, neutralizing antibody titers, and cellular responses gradually declined, reflecting the natural waning of postvaccination immunity. Nevertheless, negative results across all three assays were observed in less than 0.7% of 1181 blood samples, suggesting a persistent immune response throughout the observation period (February 2021 to December 2023).

These findings highlight the durability of immune memory and support data-driven optimization of booster dose timing to sustain long-term protection in the population.

## Figures and Tables

**Table 1:** Comparison of immune responses following COVID-19 vaccination between individuals with and without prior COVID-19 infection.

Parameter	COVID-19 Before Vaccination	*n*	Median	IQR	*p*-Value ^1^
IgG [PI]	yes	26	7.51	6.84–8.71	0.131
	no	123	7.05	6.62–8.18	
VNT [reciprocal serum dilution]	yes	26	2560	1120–2560	<0.001
	no	123	640	320–1280	
IGRA [mIU/mL]	yes	26	2500	2479–2500	0.080
	no	121	2500	1786–2500	

^1^ Mann–Whitney test, *n*-count, IQR—interquartile range (25th and 75th percentile), PI—positivity index. Note: All individuals received COVID-19 vaccination. Grouping is based on prior COVID-19 infection status before vaccination. Sampling was performed between weeks 2 and 6. Two IGRA test samples were excluded from the analysis because of preanalytical processing errors.

**Table 2:** Evaluation of IgG, neutralizing antibodies (VNT), and cellular immune response (IGRA) at defined time points after COVID-19 vaccination in individuals without prior COVID-19 infection or booster dose.

Parameter	Sampling Interval from Two Vaccination Doses	2nd to 6th Week	15th to 20th Week	27th to 32nd Week	40th to 48th Week	67th to 72nd Week
IgG [PI]	*n*	20	17	18	14	10
	median	6.8	7.3	5.1	5.1	10.0
	IQR	6.5–9.2	6.3–8.2	3.7–6.3	3.0–8.5	9.5–11.0
	*p*-value ^1^		0.182	0.016	0.017	0.084
VNT titer [reciprocal serum dilution]	*n*	20	17	18	14	10
	median	640.0	160.0	80.0	120.0	2560.0
	IQR	320–640	160–320	40–160	40–1280	1280–2560
	*p*-value ^1^		0.002	0.010	0.999	0.043
IGRA [mIU/mL]	*n*	20	14	14	14	9
	median	2500.0	1050.0	1770.0	1684.0	2500.0
	IQR	2317–2500	511–2195	674–2500	788–2500	2058–2500
	*p*-value ^1^		<0.001	0.001	0.002	0.999

^1^ Mann–Whitney test, PI—positivity index, *n*-count, IQR—interquartile range (25th and 75th percentile).

**Table 3:** Comparison of IgG levels, neutralizing antibody titers (VNT), and cellular immune response (IGRA) between individuals with and without postvaccination COVID-19 infection across defined time intervals.

	COVID-19	No			Yes			
Parameter	Sampling Interval from Two Vaccination Doses	*n*	Median	IQR	*n*	Median	IQR	*p*-Value ^1^
IgG [PI]	2nd to 6th week	20	6.8	6.5–9.2	21	7.2	6.7–7.7	0.990
	15th to 20th week	17	7.3	6.3–8.2	21	6.7	5.9–7.8	0.322
	27th to 32nd week	18	5.1	3.7–6.3	19	4.7	3.4–5.5	0.564
VNT titer [reciprocal serum dilution]	2nd to 6th week	20	640	320–640	21	320	320–640	0.125
	15th to 20th week	17	160	160–320	21	160	160–160	0.211
	27th to 32nd week	18	80	40–160	19	80	40–80	0.351
IGRA [mIU/mL]	2nd to 6th week	20	2500	2317–2500	21	2500	1583–2500	0.496
	15th to 20th week	14	1050	511–2196	21	829	655–2497	0.879
	27th to 32nd week	14	1770	674–2500	14	968	606–2500	0.546

^1^ Mann–Whitney test, *n*-count, IQR—interquartile range (25th and 75th percentile), PI—positivity index.

**Table 4:** Comparison of IgG, neutralizing antibody (VNT), and cellular immune response (IGRA) levels in individuals with different combinations of vaccination and COVID-19 infection.

Parameter	Interval: Event *—Sampling	2 Doses of Vaccination			1 Booster Dose			2 Doses of Vaccination and COVID-19 Infection			2 Doses of Vaccination, 1 Booster Dose and COVID-19 Infection			
	Week	*n*	Median	IQR	*n*	Median	IQR	*n*	Median	IQR	*n*	Median	IQR	*p*-Value ^1^
IgG [PI]	2nd to 7th	122	7.1	6.6–8.2	60	10.0	9.0–10.7	20	10.1	9.1–10.9	26	10.8	9.4–13.3	<0.001
	12th to 21st	119	7.0	5.9–8.0	56	9.4	8.3–10.3	20	9.5	8.4–11.2	23	11.9	11.2–14.0	<0.001
	25th to 32nd	119	4.8	3.6–6.1	49	10.4	9.4–11.7	18	10.7	9.4–12.2	21	11.9	10.6–13.0	<0.001
	34th to 42nd	71	3.2	2.4–5.3	36	12.0	10.8–13.5	11	13.1	10.5–13.6	16	13.6	11.8–14.4	<0.001
	43rd to 50th				6	12.3	11.2–13.0	6	13.8	12.3–17.0				0.150
	64th to 71st	9	10.1	9.7–11.0	34	14.0	10.7–17.0	12	12.2	11.0–14.8	14	11.5	10.7–12.6	0.005
	85th to 92nd				13	9.8	7.8–10.6							
	93rd to 100th	5	13.8	12.3–13.8	15	9.2	7.8–9.6	10	8.9	8.6–9.1	5	9.1	8.8–9.2	0.005
VNT titer [reciprocal serum dilution]	2nd to 7th	122	640	320–1280	60	2560	1920–2560	20	2560	2560–2560	26	>2560	2560–2560	<0.001
	12th to 21st	120	160	160–320	56	1280	960–2560	20	2560	2560–2560	23	2560	1280–2560	<0.001
	25th to 32nd	122	80	40–160	49	2560	640–2560	18	2560	640–2560	21	1280	1280–2560	<0.001
	34th to 42nd	71	80	20–160	36	2560	1280–2560	11	1280	320–2560	16	1920	640–2560	<0.001
	43rd to 50th				6	2560	1280–2560	6	2560	1280–2560				0.936
	64th to 71st	9	2560	2560–2560	34	640	320–2560	12	320	240–1280	14	960	320–2560	0.006
	85th to 92nd				13	320	320–2560							
	93rd to 100th	5	320	160–2560	15	320	320–640	10	640	320–640	5	1280	640–2560	0.672
IGRA [mIU/mL]	2nd to 7th	121	2500	1793–2500	60	2500	2266–2500	20	2500	2500–2500	26	2500	1897–2500	0.337
	12th to 21st	110	975	490–2026	55	1838	961–2287	20	2103	1427–2500	23	1234	886–2180	<0.001
	25th to 32nd	90	705	460–2060	47	1928	1129–2500	18	2438	1860–2500	22	1556.5	839–2500	<0.001
	34th to 42nd	71	760	359–1943	35	1780	1135–2500	11	2290	1518–2500	16	1306.5	804–1724	0.785
	43rd to 50th				5	1620	1139–2500	5	1687	1621–1928				
	64th to 71st	8	2500	1762–2500	32	2500	1575–2500	12	2500	1557–2500	14	1382	808–2286	0.111
	85th to 92nd				13	1273	955–2500							
	93rd to 100th				15	1551	954–2500	9	1762	1438–2500	5	1510	1063–1603	0.842

^1^ Mann–Whitney test, *n*-count, IQR—interquartile range (25th and 75th percentile), PI—positivity index, * after each event (vaccination, booster, infection) the sample collection schedule was reset (1, 3, 6, 12, 18, and 24 months; in the event of infection, a sample was obtained 3 weeks after recovery and 2 weeks after vaccination).

## Data Availability

The data presented in this study are available on request from the corresponding author. The data are not publicly available due to privacy and ethical restrictions.

## Supplementary Materials

The following supporting information can be downloaded at: https://www.mdpi.com/article/10.3390/vaccines13111163/s1, Figure S1: Timeline of COVID-19 infections, vaccinations, and sample collections among study participants. The table shows the number of respondents according to the order and timing of sample collection.

## Abbreviations

The following abbreviations are used in this manuscript:

Ig	Immunoglobulin
VNT	Virus neutralization test
SARS-CoV-2	Severe Acute Respiratory Syndrome Coronavirus 2
IFN	Interferon
ARDS	Acute respiratory distress syndrome
ACE2	Angiotensin-converting enzyme 2
IL	Interleukin
MBL	Mannose-binding lectin
PHI	Public Health Institute
IGRA	Interferon-gamma release assay
IQR	Interquartile range
PI	Positivity Index
VOC	Variant of concern
F	Female
M	Male
y	Years
