# Supplementary material for: Two-Year Follow-Up of Humoral and Cellular Immune Responses to SARS-CoV-2 in Healthcare Professionals
Source: Vaccines (Basel). 2025 Nov 14;13(11):1163. doi: 10.3390/vaccines13111163 (PMC12656521; doi:10.3390/vaccines13111163)
Supplement: Supplementary file 1 [file vaccines-13-01163-s001.zip › vaccines-3935622-supplementary.pdf]

Supplementary Materials

Figure S1. Timeline of COVID-19 infections, vaccinations, and sample collections among study participants.

Date MM/YY	07/20	08/20	09/20	10/20	11/20	12/20	01/21	02/21	03/21	04/21	05/21	06/21	07/21	08/21	09/21	10/21	11/21	12/21
COVID-19	Alfa							Beta			Delta							
1st COVID-19	1		1	5	1	13	4	1								5	9	3
2 doses							1	85	18	40	3	1	1					
1st sampling								75	12	27	30	2	2				1	
2nd COVID-19																	1	1
1 booster dose																7	26	39
2 booster doses																		
2nd sampling											76	11	25	28	4	3		
3rd sampling												1	1	67	16	23	28	3
4th sampling															8	5	65	8
5th sampling																	15	23
6th sampling																	1	2
7th sampling																		1
8th sampling																		
9th sampling																		
10th sampling																		
11th sampling																		
12th sampling																		
13th sampling																		
Total sampling								75	12	27	106	14	28	95	28	31	110	37

Date MM/YY	01/22	02/22	03/22	04/22	05/22	06/22	07/22	08/22	09/22	10/22	11/22	12/22	01/23	02/23	03/23	04/23	05/23	06/23	07/23	08/23	09/23	10/23	11/23	12/23	n = cour
COVID-19	Omicron																								
1st COVID-19	8	13	4	1		2	1	1	3		1	1													78
2 doses																									149
1st sampling																									149
2nd COVID-19	2/1	2/1	1					1	1	1					1	1									14
1 booster dose	18	3	1		1																				95
2 booster doses											1	1										1			3
2nd sampling			1																						148
3rd sampling	4	1			1																				145
4th sampling	33	13	5		1		1	1																	140
5th sampling	16	21	16	13	11	2	4	7		1	2				1										132
6th sampling	2	14	23	8	26	12	10	6	3	3	5		3	4		2			1						125
7th sampling			3	2	13	23	5	17	11	12	6		3	4	1	1	1		1	1					105
8th sampling				1		3	3	14	18	10	7	8	2	4	7	5	4		2	3	1		1	1	94
9th sampling						1			2	3	7	4	4	7	15	6	5	4		4	5	3	2		72
10th sampling									1				2	1	4	4	5	4	1	6	8	5	5	2	48
11th sampling															1	1	1	2	1	2	4	2	4		18
12th sampling																					1	2		1	4
13th sampling																							1		1
Total sampling	55	49	48	24	52	41	23	45	35	29	27	12	14	20	29	19	16	10	6	16	19	12	13	4	1181

COVID-19 before / COVID-19 after vaccination

Initiation of vaccination

Alfa
Beta
Delta
Omicron
